# Prevalence and Correlates of Intimate Partner Violence among Women Attending Different Primary Health Centers in Aljouf Region, Saudi Arabia

**DOI:** 10.3390/ijerph19010598

**Published:** 2022-01-05

**Authors:** Doaa M. Abdel-Salam, Bashayer ALruwaili, Doaa Mohamed Osman, Maha Mamluh M. Alazmi, Sama Ayman Mater ALghayyadh, Rawan Ghazi Zaki Al-sharari, Rehab A. Mohamed

**Affiliations:** 1 Public Health and Community Medicine Department, Faculty of Medicine, Assiut University, Assiut 71526, Egypt; Doaamouhammed@aun.edu.eg; 2 Family and Community Medicine Department, College of Medicine, Jouf University, Sakaka 72388, Saudi Arabia; bfalrwili@ju.edu.sa; 3 College of Medicine, Jouf University, Sakaka 72388, Saudi Arabia; Mahaalazmi000@gmail.com (M.M.M.A.); Sama.almater@gmail.com (S.A.M.A.); Rawan20ghazi@gmail.com (R.G.Z.A.-s.); 4 Family Medicine Department, Faculty of Medicine, Suez Canal University, Ismailia 41522, Egypt; rehabali@med.suez.edu.eg

**Keywords:** intimate partner violence, prevalence, associated factors, Saudi women

## Abstract

Background and Objectives: Intimate partner violence (IPV) is a serious and widespread problem worldwide. IPV can seriously influence the physical, mental, sexual, and reproductive health of women as well as the welfare of their children. In the Middle East, IPV is pervasive and widely acceptable. The present study was done to determine the prevalence and correlates of IPV among women attending different primary health centers in the Aljouf region, Saudi Arabia. Methods: A cross-sectional study was conducted among 403 Saudi women attending different primary health centers in the Aljouf region, Saudi Arabia. A structured anonymous questionnaire was distributed to the targeted population during a face-to-face interview. Data analysis was done using the SPSS program, version 24. Results: The present study showed that 30.3% of the participants had been exposed to IPV over the last year. Concerning the types of violence, the present study revealed that emotional violence is the highest followed by physical and then sexual violence representing 92.6%, 67.2%, and 44.3%, respectively. The significant predictors of IPV were women with one to three children (OR = 7.322, *p*-value = 0.006), women with four children or more (OR = 13.463, *p*-value = 0.006), and women married to husbands with aggressive behavior (OR = 98.703, *p*-value < 0.001). Not taking the approval on marriage was significantly associated with more exposure to violence (OR = 3.190, *p*-value = 0.042). In addition, husband smoking status was a significant predictor for IPV (OR = 2.774, *p*-value = 0.012). However, women married to alcoholic drinkers had a significantly lower risk for exposure to IPV (OR = 0.108, *p*-value = 0.040). On the other hand, women’s age, marital status, women’s educational level, monthly income in RS, perception of income sufficiency, marriage duration, the age difference between women and their husband, and drug abuse status of the husband were not significant predictors of IPV (*p*-value ≥ 0.05). Sociocultural effects were the most frequent reason for IPV as reported by the participants (57.4%). The most common consequences of IPV were psychological problems (75.4%) and injuries (42.6%). Women’s reactions to IPV were leaving home (32.8%) or no reaction (36.8%) to retain their marriage. Conclusions: IPV remains an important public health problem among married women in this study area. Urgent interventions including educational and screening programs for Saudi women are required to mitigate the problem.

## 1. Introduction

Intimate partner violence (IPV) is defined as any attitude or behavior by an intimate partner that results in physical, sexual, or psychological consequences including physical aggression, sexual compulsion, and psychological abuse [1]. IPV against women is identified as an important public health problem including a serious violation of women’s human rights [2]. IPV can negatively influence the mental, physical, sexual, and reproductive health of women and their children [3]. 

There is a huge difference in the prevalence of IPV towards women in different countries [4]. The World Health Organization (WHO) showed that nearly one in three or 30% of women have been exposed to physical and/or sexual violence by an intimate partner according to the analysis of data from 2000 to 2018 among 161 countries [1]. WHO reported that 29.8% of women in the United States and 25.4% in European countries have been exposed to physical or sexual violence by an intimate partner [5]. In addition, one out of three women in Egypt, Palestine, Tunisia, and Israel have been exposed to violence in 2003–2005 [6,7]. 

Several sociodemographic characteristics have been associated with IPV against women. These sociodemographic characteristics include low education, poor working status, poor financial status, child maltreatment, family troubles, and alcohol or drug use [8,9]. 

In the Middle East, IPV is similarly pervasive and widely acceptable [4]. IPV is widely prevalent and acceptable in Saudi Arabia [10,11,12]. Saudi Arabia is considered a closed community because little was known about their reproductive health issues. The willingness of Saudi women to participate in studies discussing reproductive health needs and exploring their privacy is limited. It is well known that the man in Saudi society is the master of the family and women are responsible for taking care of the family avoiding family destruction irrespective of their happiness. Saudi norms prevent women from asking for assistance from strangers or reporting their reproductive health issues to physicians or families. Saudi women are reluctant to disclose being exposed to IPV to save her family from destruction. With Saudi Arabia’s Vision 2030, different Saudi norms have been changed and there is an improvement in women’s human rights as Saudi women become empowered. 

This study will provide a snapshot of the experienced IPV among Saudi women and their correlates and consequently help the policymakers in designing interventions including educational and screening programs for women attending different primary health centers. This will be reflected in lowering the prevalence of IPV among Saudi women. 

## 2. Participants and Methodology

### 2.1. Study Design and Setting 

A cross-sectional study was accomplished to investigate the prevalence and correlates of IPV among Saudi women attending different primary health centers in the Aljouf region, Saudi Arabia. The Aljouf region is situated in the north part of Saudi Arabia and has a population of 520,737 according to the executed census of 2018. There are four governorates in the Aljouf region: Skakka, Tabargel, Alqurayat, and Domat Al-Jandal. This study was carried out in different primary health centers of the Aljouf region. 

### 2.2. Sample Size Estimation

The sample size calculation was done using n = P (1 − P) z^2^/ d^2^ assuming the prevalence of IPV as 39% [12], Z = 1.96 and d = 0.05, and applying a confidence level of 95%. The calculated sample size was 366. The sample size was raised to 403 after adding 10% as a non-response rate.

### 2.3. Sampling Technique

**Figure 1:**
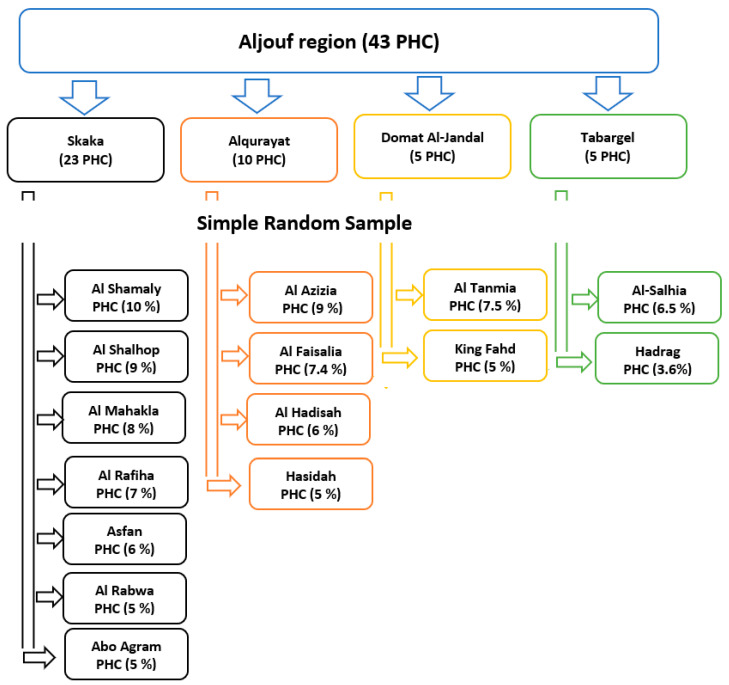
Schematic diagram of the sampling technique.

The target population was selected from primary health centers of the Aljouf region, Saudi Arabia during the study period. There were 43 primary health centers in the Aljouf region, Saudi Arabia. By simple random sampling technique, 15 centers were selected out of 43. The number of women chosen in each primary health center was proportional to the number of women served by this center until reaching the estimated sample size Figure 1. The target population of the present study was chosen from the waiting areas of the primary health centers after being informed about the objectives of the study. Data collection was continued from January to April 2021. The questionnaires were distributed by the researchers to all women of the selected centers except those who refused to participate in the study. 

### 2.4. Inclusion and Exclusion Criteria

Saudi females who were married at the time of the study or divorced/widowed less than one year before the study were included in the present study. Women with psychiatric diseases or who do not meet the above criteria were excluded from the study.

### 2.5. Data Collection Tool

In the present study, IPV is defined as violence that is inflicted on a woman by her spouse over the last year, including physical, psychological, or emotional, and sexual violence. Physical violence was identified as slapping, hitting, choking, punching, pushing, and various types of contact that lead to physical injury to the women [13,14]. Emotional violence was identified as intimidating, threatening, weakening the women’s self-esteem, and limiting the women’s freedom [13,14]. Sexual violence was identified as using the power to elicit unsafe, unwanted, or despicable sexual activity [13,14]. 

An anonymous structured questionnaire was used for data collection. The questionnaire was composed of four parts. The first part inquired into sociodemographic features of the participants such as age, marital status, educational level, employment, monthly income, age at marriage, marriage duration, having children, number of children, residence, etc. The second part of the questionnaire was about the husband’s characteristics such as education, employment, job type, smoking status, aggressive behavior, alcohol consumption, and drug abuse. The third part inquired about the exposure to IPV over the last year using the Arabic version of the Norvold Domestic Abuse Questionnaire. This questionnaire is well-validated and reliable to estimate the prevalence of different forms of IPV: emotional, physical, and sexual [15]. The alpha reliability coefficient of the Norvold Domestic Abuse Questionnaire was 0.75 [15]. The fourth part of the questionnaire consisted of questions related to the reasons, consequences, and reactions to IPV as reported by the respondents.

A pilot study was conducted on 30 eligible women to assess the clarity and face validity of the used questionnaire. No modifications were performed on the used questionnaire. Results of the pilot study were not included in the present study.

### 2.6. Statistical Analysis

Data were analyzed using the SPSS program, version 24 (SPSS Inc., Chicago, IL, USA). Descriptive statistics were utilized using number and percentage for categorical variables, mean ±SD for continuous variables. Factors associated with IPV were identified using the Chi-square test. To adjust for confounding variables, logistic regression analysis was carried out. Logistic regression was performed on those variables that were identified as significant by the Chi-square analysis. A multicollinearity test was performed. Correlation coefficient values between all the variables entered in the model were less than 0.7 and the VIF value for all variables was less than 4. Finally, *p*-value ≤ 0.05 was considered statistically significant.

### 2.7. Ethical Considerations

The proposal was submitted to the Ethical Review Committee of Jouf University, Saudi Arabia, and data collection was commenced after ethical clearance (Approval number: 04-07/41). A written consent form with a statement of confidentiality was taken from women who welcomed participation in the present study. Confidentiality of the data was confirmed.

## 3. Results

**Figure 2:**
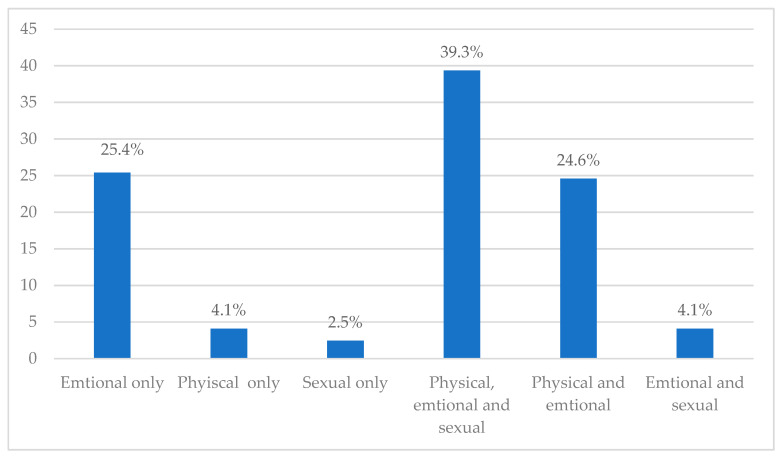
Distribution of different types of IPV among women attending different primary health centers in the Aljouf region, Saudi Arabia.

Table 1 reveals that 30.3% of the women in the present study had been exposed to IPV. Concerning the types of violence, emotional violence is the highest followed by physical and then sexual violence representing 92.6%, 67.2%, and 44.3% respectively. Figure 2 shows that 39.3% of women have been exposed to all types of violence. Nearly one quarter of women (25.4%) have been exposed to emotional violence only. Physical and emotional violence was reported by 24.6% of women. Physical and sexual violence was reported by 4.1% and 2.5% of women, respectively.

Table 2 depicts the sociodemographic correlates of IPV among women attending different primary health centers in the Aljouf region, Saudi Arabia. Increased age of women was significantly associated with more exposure to violence. Married women, women with university or postgraduate education, women with high income, and women who considered their income as sufficient were significantly more exposed to IPV compared to other women. In addition, marriage duration >10 years, having children, and having more than four children were significantly associated with more exposure to IPV. Not taking women’s approval on marriage was significantly associated with more exposure to violence. Table 2 also reveals that IPV was significantly higher when the age difference between couples was 1–10 years. Table 3 shows that the characteristics of husbands that significantly influence the exposure to IPV are being a smoker and having aggressive behavior. However, women whose husbands are alcoholic drinkers or drug abusers were significantly less exposed to violence. Table 4 demonstrated the predictors of IPV using the adjusted logistic regression model. The significant predictors of IPV were women with one to three children (OR = 7.322, *p*-value = 0.006), women with four children or more (OR = 13.463, *p*-value = 0.006), and women married to husbands with aggressive behavior (OR = 98.703, *p*-value < 0.001). Not taking the approval on marriage was significantly associated with more exposure to violence (OR = 3.190, *p*-value = 0.042). In addition, the husband’s smoking status was a significant predictor for IPV (OR = 2.774, *p*-value = 0.012). However, women married to alcoholic drinkers had a significantly lower risk for exposure to IPV (OR = 0.108, *p*-value = 0.040). On the other hand, the women’s age, marital status, educational level, monthly income in RS, perception of income sufficiency, marriage duration, the age difference between women and their husband, and drug abuse status of the husband were not significant predictors of IPV (*p*-value ≥ 0.05). Table 5 investigates the reasons, consequences, and reactions to IPV as reported by the participants. The most frequent reason for IPV was the sociocultural effects (57.4%). Concerning the frequency of IPV, 13.1% and 36.1% of the women were exposed to violence once a day and once a month, respectively. Most of the participants reported that psychological problems (75.4%) and injuries (42.6%) were the residual influences of violence. Regarding the reactions to violence, 36.8% of the women do nothing and 32.8% leave their homes.

## 4. Discussion

IPV is a serious, rising, and preventable public health problem that affects millions of people worldwide. IPV has become a major topic in Saudi Arabia, with official and nongovernmental organizations analyzing them from both social and medical viewpoints [16]. The prevalence of IPV varies per country, based on cultural taboos as well as how violence is defined [8]. Even though IPV is widely acceptable in many countries, it is nonetheless considered a breach of women’s rights. The significance of violence arises from the fact that it has an impact on both men and women, as well as children. Witnessing abuse as a child is a well-known risk factor for their future engagement in violence [17]. The present study was done to determine the prevalence and correlates of IPV among women attending different primary health centers in the Aljouf region, Saudi Arabia. The present study revealed that the prevalence of IPV was 30.3%, which is higher than that reported among female visitors to primary care centers in Riyadh, Saudi Arabia, where the prevalence was 20% [10]. Another study conducted among Omani women demonstrated that the prevalence of violence was 28.8% [18]. The present study results were nearly consistent with other studies conducted in Saudi Arabia, where the prevalence of IPV was 32% among women from 13 governorates in the kingdom [19] and 33.24% among women in the western region [8]. However, a much higher prevalence of IPV was reported in a study conducted among women in Central Ethiopia, where 77% and 62.4% of the participants showed that they have been exposed to IPV in their lifetime and the last one year, respectively [20]. In addition, the prevalence of IPV in the present study was much lower than that reported among women in Iraq, where the prevalence of the overall lifetime and the overall past-year IPV against women was 58.6% and 45.3%, respectively [21]. Another study conducted in Bangladesh revealed that 87% of the women have been exposed to IPV [22]. Violence was reported by 41.6% of women in Sudan [23]. The prevalence of lifetime IPV and violence occurring within the year before the study was 43% and 26% among Chinese women [24]. These differences in the prevalence of IPV could be attributed to sociodemographic and cultural characteristics of each study population. In addition, different cultures have different perspectives on violence in general and the nature of husband–wife interactions in particular. In some communities, what is deemed violence in one culture may be regarded as acceptable in another. As a result, each community has its perspective on the problem.

Emotional violence has a more complex definition, and its perception may alter among cultures. Many husbands and even wives in Muslim societies may see violence against women as a justified punishment for the wife’s misconduct [25]. The current study showed that emotional violence is the highest, followed by physical and then sexual violence representing 92.6%, 67.2%, and 44.3%, respectively. This finding is consistent with several studies carried out locally or internationally [8,12,18]. A recent study conducted in Iran revealed that emotional violence was most common, with more than half of the women complaining about it [26]. The reasons for the predominance of emotional violence could be attributed to the fact that all other types of violence either physical or sexual may have an underlying emotional component [27]. Moreover, emotional violence is often considered a precursor to physical violence [28].

Another study in Uganda concluded that almost an equal number of women experienced both physical and emotional forms of IPV (41% and 40%, respectively), while sexual IPV was the least common [29]. In China, of the respondents who were physically abused in their lifetime, 29% were also sexually abused by their partners [24].

The socio-demographic factors that were independently associated with IPV in the current study were consistent with earlier studies with a few variations. In the current study, more education of women was associated with more exposure to IPV. This has been recorded previously in Arab women and was linked to educated women’s propensity to challenge male dominance in a male-dominated society [30]. Many studies reported that women who had higher education were at risk of IPV [10,31,32]. However, other studies found that women with more years of education were less likely to be at risk of violence [33,34]. The difference of reported IPV among different studies because of education may be related to different cultures and educational levels between study populations. However, men’s sense of self as family heads, decision-makers, and earners may be threatened by educated women [35]. Some males may perceive economically independent and educated female partners as intimidating and, therefore, IPV may be used as part of a threatened man’s strategy to maintain control over his partner [36].

Previous WHO reports indicated that one of the most consistent factors associated with a man’s increased likelihood of committing violence against a partner was young age [37]. Many studies showed that the prevalence of IPV decreases with increasing women’s age [10,11,38]. However, the present study revealed that violence increases with increasing women’s age. This may be contributed to low help-seeking behavior among old Saudi women because of the feeling of guilt, shame, and embarrassment. Thus, they may accept violence and its continuation.

Poor or insufficient income may result in family pressure, dissatisfaction, and inadequacy, creating an environment conducive to violence [39,40]. However, in the current study, women with high income and women who considered their income as sufficient were significantly exposed to violence compared to other women. This difference might be because of socioeconomic differences between the countries, and most families have sufficient income in Saudi Arabia.

Women who had been in a relationship for a long time had a higher risk of suffering violence than women who had been in a relationship for a shorter time [29]. This finding is in concordance with the current study. However, this conclusion contradicts Urquia et al study, which stated that only women with less marital duration suffered higher violence than women with long marital duration [41]. However, women in Saudi Arabian society usually decide to stay in the relationship for the sake of children and traditions even if she was abused [8].

In the present study, women who have children were more likely to be exposed to IPV than other women. The present study also revealed that an increasing number of children was associated with more exposure to violence. This finding was consistent with other studies [20,42]. This may be because having many children can raise conflicts and stress and result in economic and social problems within the family and consequently raise the risk of violence.

The current study indicates that the husband’s characteristics that significantly influence the exposure to IPV are being a smoker and having aggressive behavior. This evidence points to the dominance of men in Saudi Arabian society, with wives expected to obey their husbands and accept everything as normal male conduct [43,44]. Similar findings have been reported in Uganda where most women (71%) experienced partner controlling behaviors [29]. In addition, a study conducted in Egypt showed that smoking and drug use habits among husbands were significantly associated with spousal violence [40].

Furthermore, alcohol and drug use among the husbands, which was correlated with IPV in many studies [20,40] and even reported by women in the present study as reasons of violence, were not associated with IPV in the current study, probably due to the small sample size (drug abuse and alcoholism are not prevalent in the Saudi population).

Taking women’s approval on marriage is considered a human right and will significantly influence the exposure to IPV. The present study revealed that not taking women’s approval on marriage is significantly associated with more exposure to violence. This finding was consistent with a study conducted in Turkey to investigate risk factors of domestic violence [45]. This can be explained by the fact that not taking women’s approval on marriage is associated with the economic independence of these women and thus they accept violence in all its forms.

In the present study, most women thought that socio-cultural influences were the main reasons for IPV against women. This finding is consistent with a study conducted in Riyadh, Saudi Arabia [10]. Kury et al. reported that traditional attitudes toward women and a general tolerance of violent behavior were the most common causes of IPV in European countries, which is consistent with this study’s findings [46].

Women who are assaulted by their partners are more likely to experience despair, anxiety, and phobias than those who are not abused. The most common residual effects of IPV in the current study were psychological problems. In the WHO multi-country study, women who had ever encountered any violence reported significantly more psychological trauma, thoughts of suicide, and attempted suicide than those who had not [37]. The majority of women in this study complained of injuries as residual effects of IPV. According to a survey performed in Saudi Arabia, 53% of husbands who physically abused their spouses believed it was their responsibility to correct their wives’ behavior, even if it meant physical injuries [44].

In the current study, women’s reactions to violence were leaving home or no reaction to retain their marriage. In the Arab countries, nearly half of women were found to tolerate IPV and not seek any assistance from any legal or social institutions or ever healthcare professionals [12]. Possible causes may include being raised in a conservative society and fear of humiliation, normalizing various forms of abuse in an overwhelmingly male culture, or a lack of knowledge about where to seek help [8]. 

The present study has some strengths such as using a standardized tool for assessing violence and examining several risk factors including culturally specific ones in a relatively large sample.

However, there were some limitations. For example, the cross-sectional design in this study limited the detection of the temporal relationship between risk factors and violence. The sensitivity of some questions in the questionnaire is another limitation because they address sensitive issues like sexual violence. Thus, some participants may hesitate to answer these questions.

## 5. Conclusions

The present study is among few studies done in the Middle East regarding IPV and adds supplementary data to the literature about this closed society. In the present study, Saudi women complained of different forms of IPV. The current study shows that emotional violence is the highest form, followed by physical and then sexual violence. Sociocultural effects were the most frequent reason for IPV as reported by the participants. Thus, addressing this public health problem is of urgent importance and requires collaborations between multiple sectors including policymakers, professionals, and stakeholders to mitigate this situation. A sociocultural intervention is recommended to empower women through educational programs, especially about the value of women’s approval before marriage and the role of family planning. Moreover, future studies are needed to investigate aspects of male control in intimate relationships.

## Figures and Tables

**Table 1:** Prevalence and types of IPV among women attending different primary health centers in the Aljouf region, Saudi Arabia.

Variable	No. (%)
Exposure to IPV	
Yes	122 (30.3%)
No	281 (69.7%)
Types of IPV ^1^	
Emotional	113 (92.6%)
Physical	82 (67.2%)
Sexual	54 (44.3%)

^1^ More than one answer had been reported.

**Table 2:** Sociodemographic correlates of IPV among women attending different primary health centers in the Aljouf region, Saudi Arabia.

Sociodemographic Features		Exposure to IPV		*p*-Value
	Total	Exposed (n = 122) No. (%)	Non-Exposed (n = 281) No. (%)	
Age				*p* < 0.001
≤20	33 (8.2%)	9 (7.4%)	24 (8.5%)	
21–30	189 (46.9%)	33 (27.0%)	156 (55.5%)	
31–40	87 (21.6%)	36 (29.5%)	51 (18.1%)	
≥41	94 (23.3%)	44 (36.1%)	50 (17.8%)	
Mean ± SD	32.73 ± 2.12			
Marital status				*p* < 0.001
Married	368 (91.3%)	98 (80.3%)	270 (96.1%)	
Divorced/widowed	35 (8.7%)	24 (19.7%)	11 (3.9%)	
Educational level				0.007
Illiterate	7 (1.8%)	5 (4.1%)	2 (0.7%)	
Primary/preparatory	11 (2.7%)	7 (5.7%)	4 (1.4%)	
Secondary/diploma	81(20.1%)	22 (18.0%)	59 (21.0%)	
University/postgraduate	304 (75.4%)	88 (72.1%)	216 (76.9%)	
Employment				0.192
Employed	185 (45.9%)	62 (50.8%)	123 (43.8%)	
Unemployed	218 (54.1%)	60 (49.2%)	158 (56.2%)	
Monthly income				*p* < 0.001
<5000 RS ^1^	86 (21.3%)	30 (24.6%)	56 (19.9%)	
5000–7000 RS	80 (19.9%)	9 (7.4%)	71 (25.3%)	
>7000 RS	237 (58.8%)	83 (68.0%)	154 (54.8%)	
Monthly income sufficient				*p* < 0.001
Yes	279 (69.2%)	67 (54.9%)	212 (75.4%)	
No	124 (30.8%)	55 (45.1%)	69 (24.6%)	
Age at marriage				0.192
<20	88 (21.8%)	37 (30.3%)	51(18.1%)	
≥20	315 (78.2%)	85 (69.7%)	230 (81.9%)	
Mean ± SD	22.39 ± 4.38			
Marriage duration				*p* < 0.001
0–5 years	168 (41.7%)	34 (27.9%)	134 (47.7%)	
6–10 years	55 (13.6%)	13 (10.7%)	42 (14.9%)	
>10 years	180 (44.7%)	75 (61.5%)	105 (37.4%)	
Having children				*p* < 0.001
Yes	319 (79.2%)	110 (90.2%)	209 (74.4%)	
No	84 (20.8 %)	12 (9.8%)	72 (25.6%)	
Number of children				0.008
1–3	169 (41.9%)	47 (42.7%)	122 (58.4%)	
≥4	150 (37.2%)	63 (57.3%)	87 (41.6%)	
Residence				0.455
House of husband	349 (86.6%)	108 (88.5%)	241(85.8%)	
House of Husband’s family	54 (13.4%)	14 (11.5%)	40 (14.2%)	
Relative relationship with your husband				0.198
Yes	168 (41.7%)	45 (36.9%)	123 (43.8%)	
No	235 (58.3%)	77 (63.1%)	158 (56.2%)	
Approval on marriage was taken				*p* < 0.001
Yes	61 (15.1%)	40 (32.8%)	21 (7.5%)	
No	342 (84.9%)	82 (67.2%)	260 (92.5%)	
The age difference between you and your husband				0.015
No difference	54 (13.4%)	8 (6.5%)	46 (16.4%)	
1–10 years	315 (78.2%)	100 (82.0%)	215 (76.5%)	
>10 years	34 (8.4%)	14 (11.5%)	20 (7.1%)	

^1^ RS: Saudi Riyal.

**Table 3:** Relationship between IPV and characteristics of husbands of women attending different primary health centers in Aljouf region, Saudi Arabia.

Husbands’ Characteristics		Exposure to IPV		*p*-Value
	Total	Exposed (n = 122) No. (%)	Non-Exposed (n = 281) No. (%)	
Husband education				0.309
Illiterate	6 (1.5%)	3 (2.4%)	3 (1.1%)	
Primary/preparatory	25 (6.2%)	10 (8.2%)	15 (5.3%)	
Secondary/diploma	100 (24.8%)	25 (20.5%)	75 (26.7%)	
University/postgraduate	272 (67.5%)	84 (68.9%)	188 (66.9%)	
Husband Employment				0.082
Working	322 (79.9%)	94 (77.0%)	228 (81.1%)	
No working	29 (7.2%)	6 (4.9%)	23 (8.2%)	
Retired	52 (12.9%)	22 (18.1%)	30 (10.7%)	
Job type				0.427
Civil	266 (66.0%)	84 (68.9%)	182 (64.8%)	
Military	137 (34.0%)	38 (31.1%)	99 (35.2%)	
Smoking status				*p* < 0.001
Yes	173 (42.9%)	73 (59.8%)	100 (35.6%)	
No	230 (57.1%)	49 (40.2%)	181(64.4%)	
Aggressive behavior				*p* < 0.001
Yes	109 (27.0%)	97 (79.5%)	12 (4.3%)	
No	294 (73.0%)	25 (20.5%)	269 (95.7%)	
Alcohol drinking				*p* < 0.001
Yes	27 (6.7%)	22 (18.0%)	5 (1.8%)	
No	376 (93.3%)	100 (82.0%)	276 (98.2%)	
Drug abuse				*p* < 0.001
Yes	24 (6.0%)	20 (16.4%)	4 (1.4%)	
No	379 (94.0%)	102 (83.6%)	277 (98.6%)	

**Table 4:** Logistic regression revealing the predictors of IPV among women attending different primary health centers in Aljouf region, Saudi Arabia.

	Adjusted Regression Model			
	OR	95% C.I.		*p*-Value
		Upper	Lower	
Women age (in years)	1.005	0.948	1.065	0.873
Marital status (Married)	Reference Group			
Divorced/widowed	3.345	0.823	13.587	0.091
Women educational level (University/postgraduate)	Reference Group			
Less than university	1.011	0.395	2.586	0.983
Monthly income > 7000 RS	Reference Group			
≤7000 RS	0.431	0.163	1.137	0.089
Monthly income sufficient (Yes)	Reference Group			
No	1.378	0.562	3.378	0.484
Marriage duration (0–5 years)	Reference Group			
6–10 years	0.645	0.183	2.266	0.494
More than 10 years	0.326	0.081	1.318	0.116
Number of children (No children)	Reference Group			
One to three children	7.322	1.776	30.177	0.006
Four children or more	13.463	2.126	85.256	0.006
Approval on marriage was taken (Yes)	Reference Group			
No	3.190	1.042	9.767	0.042
The age difference between you and your husband (No difference)	Reference group			
1–10 years	2.978	0.840	10.558	0.091
More than 10 years	2.624	0.478	14.410	0.267
Husband smoking status (No)	Reference group			
Yes	2.774	1.246	6.175	0.012
Husband aggressive behavior (No)	Reference group			
Yes	98.703	37.881	257.179	*p* < 0.001
Husband alcohol drinking (No)	Reference group			
Yes	0.108	0.013	0.904	0.040
Husband drug abuse (No)	Reference group			
Yes	3.589	0.445	28.929	0.230

The logistic regression model was statistically significant, χ^2^ = 279.86, *p* < 0.001. The model explained 70.8% (Nagelkerke R2) of the variance in IPV exposure and correctly classified 90.6% of cases.

**Table 5:** Reasons, consequences, and reactions to IPV among women attending different primary health centers in the Aljouf region, Saudi Arabia.

	No. (%) (n = 122)
Causes of IPV as reported by women ^1^	
Sociocultural effects	70 (57.4%)
Insufficient income	35 (28.7%)
Jealousy	28 (23.0%)
Alcohol abuse	22 (18 %)
Stressors	21 (17.2%)
Drug abuse	20 (16.4%)
Treachery	20 (16.4%)
Frequency of IPV	
Once/day	16 (13.1%)
Once/week	44 (36.1%)
Once or more/month	62 (50.8%)
Residual influences of IPV ^1^	
Psychological problems	92 (75.4%)
Injuries	52 (42.6%)
No effects	17 (13.9%)
Hospital admission	12 (9.8%)
Taking drugs	12 (9.8%)
Medical problems	3 (2.5%)
Reactions to IPV	
No reaction	45 (36.8%)
Leave home	40 (32.8%)
Request divorce	23 (18.9%)
Go to doctor	9 (7.4%)
Call police	5 (4.1%)

^1^ More than one answer had been reported.

## Data Availability

Data are available from the corresponding author upon request.
